# Persistent Hypercoagulability After Radical Prostatectomy: Biomarker Dynamics and Implications for Individualized Thromboprophylaxis

**DOI:** 10.3390/jcm15124743

**Published:** 2026-06-18

**Authors:** Matyas Benyo, Marie Al-Muhanna, Zsuzsanna Molnar, Janos Docs, Tamas Takacs, Jolan Harsfalvi

**Affiliations:** 1 FertiMed LP Outpatient Center, 4027 Debrecen, Hungary; info@fertimed.hu; 2 Department of Pediatrics, Faculty of General Medicine, University of Debrecen, 4032 Debrecen, Hungary; al-muhanna.marie@med.unideb.hu; 3 Department of Urology, Faculty of General Medicine, University of Debrecen, 4032 Debrecen, Hungary; docs.janos@med.unideb.hu; 4 Centre of Assisted Reproduction, Department of Obstetrics and Gynaecology, Semmelweis University, 1097 Budapest, Hungary; takacs.tamas@semmelweis.hu; 5 Department of Biophysics and Radiation Biology, Faculty of General Medicine, Semmelweis University, 1094 Budapest, Hungary; harsfalvi.jolan@semmelweis.hu

**Keywords:** venous thromboembolism, postoperative period, thrombin generation, von Willebrand factor, endothelial activation, risk stratification, extended thromboprophylaxis, perioperative care

## Abstract

Venous thromboembolism (VTE) remains a clinically relevant complication of radical prostatectomy despite advances in surgical techniques and perioperative care. Current thromboprophylaxis strategies are largely based on fixed-duration approaches and static risk models focused on the early postoperative period. However, accumulating evidence suggests that postoperative hypercoagulability is a dynamic and prolonged process that may extend beyond this timeframe. This review summarizes the pathophysiological mechanisms and temporal dynamics of postoperative hypercoagulability after radical prostatectomy, with particular emphasis on biomarker-based evidence, including thrombin generation and von Willebrand factor. Clinical and laboratory findings suggest that haemostatic activation may persist after hospital discharge, supporting the concept of a biologically relevant post-discharge period during which insufficiently captured thrombotic risk may remain despite apparent clinical recovery. Current risk assessment models do not account for the time-dependent nature of postoperative haemostatic changes and do not incorporate biomarker data. This discrepancy highlights a gap between guideline-based thromboprophylaxis strategies and the underlying biological processes. To address this, we propose a conceptual framework in which postoperative thromboprophylaxis is considered in relation to the temporal evolution of hypercoagulability. This framework is hypothesis-generating and may help inform future studies aimed at identifying patients who could benefit from extended prophylaxis while avoiding unnecessary anticoagulation in those with more rapid haemostatic recovery. Further prospective studies are required to validate biomarker-guided strategies and to define clinically actionable thresholds for individualized thromboprophylaxis in prostate cancer patients undergoing radical prostatectomy.

## 1. Introduction

Venous thromboembolism (VTE) is a major cause of morbidity and mortality in surgical patients, particularly in the setting of oncological procedures [1,2,3,4]. Prostate cancer (CaP) is among the most common malignancies in men, and both the disease itself and its treatment are associated with an increased risk of thromboembolic events [5]. Radical prostatectomy (RP), although a standard curative treatment for localized disease, is associated with a non-negligible thrombotic risk, with reported VTE incidence varying according to patient characteristics, surgical technique, and prophylactic strategies [6,7].

Urological guideline recommendations, such as those from the European Association of Urology and the American Urological Association, primarily focus on the perioperative period and emphasize the balance between thrombotic and bleeding risks [8,9,10]. Although these guidelines acknowledge that thrombotic risk may persist for weeks after surgery, they are based on limited procedure-specific evidence and in urology often rely on extrapolation from other surgical settings [8,9,10]. More recent European guidance has also emphasized that for many urological procedures, the balance between thrombotic benefit and bleeding harm is procedure-specific and patient-specific, favouring a more tailored approach rather than a single uniform recommendation [8]. Nevertheless, these approaches do not fully incorporate the dynamic changes in the haemostatic system after RP. In addition, the lack of long-term follow-up of haemostatic changes, together with the observation that thromboembolic events frequently occur after hospital discharge, leads to an incomplete understanding of persistent postoperative hypercoagulability [11,12,13,14]. Direct biomarker-based evidence in the radical prostatectomy setting remains limited and is derived mainly from a small number of longitudinal observational studies.

Emerging evidence indicates that radical prostatectomy induces sustained alterations in both primary and secondary haemostasis, including endothelial activation, increased levels and functional changes in von Willebrand factor, and enhanced thrombin-generating potential [12,13,15]. These findings suggest that postoperative hypercoagulability may extend beyond the conventionally defined perioperative window. However, the available radical prostatectomy-specific biomarker evidence remains limited, and much of it is based on small observational studies rather than clinically validated predictive models. Importantly, the temporal relationship between biomarker dynamics and clinical thrombotic risk remains insufficiently characterized.

In this review, we examine the pathophysiological mechanisms and available clinical evidence underlying postoperative hypercoagulability after RP and discuss the implications for thromboprophylaxis. We propose that persistent postoperative hypercoagulability may represent a biologically relevant phase after radical prostatectomy that has not been sufficiently clinically characterized. RP may therefore serve as a useful clinical model in which cancer-related and surgery-related prothrombotic mechanisms, as well as their postoperative biomarker correlates, can be studied within a relatively standardized operative setting.

### Literature Search Strategy and Review Approach

This article was designed as a focused narrative review rather than a systematic review or meta-analysis. The literature base was originally developed from an EndNote library initiated in 2011 during earlier work on thrombin generation and haemostatic biomarker changes after radical prostatectomy. This library was subsequently updated during related research on postoperative haemostatic activation, endothelial perturbation, von Willebrand factor, and thromboprophylaxis in prostate cancer surgery. For the present manuscript, this pre-existing literature base was supplemented by a structured updated database search to improve transparency, reproducibility, and coverage of the relevant literature.

The updated structured search was conducted in four bibliographic databases: PubMed/MEDLINE, Web of Science Core Collection, Embase, and Scopus. Google Scholar was used only as a supplementary source for citation tracking and identification of additional potentially relevant records. Reference lists of key articles, reviews, and guidelines were also screened manually. The final structured database search and EndNote-based deduplication were completed on 10 June 2026. No formal date restriction was applied during the database search. However, during manuscript revision, particular attention was given to recent evidence, while older studies were retained when they were considered methodologically, mechanistically, or historically important for the interpretation of postoperative haemostatic changes and thromboprophylaxis after radical prostatectomy.

The search strategy focused on publications related to radical prostatectomy, prostate cancer surgery, postoperative hypercoagulability, venous thromboembolism, thromboprophylaxis, thrombin generation, von Willebrand factor, endothelial activation, D-dimer, fibrinogen, and postoperative biomarker dynamics. The following search terms and their combinations were used and adapted according to the syntax of each database: “radical prostatectomy” OR “prostate cancer surgery” combined with “venous thromboembolism”, “deep vein thrombosis”, “pulmonary embolism”, “thromboprophylaxis”, “hypercoagulability”, “thrombin generation”, “von Willebrand factor”, “endothelial activation”, “D-dimer”, and “fibrinogen”, using Boolean operators AND and OR. A representative search string was: (“radical prostatectomy” OR “prostate cancer surgery”) AND (“venous thromboembolism” OR “deep vein thrombosis” OR “pulmonary embolism” OR thromboprophylaxis OR hypercoagulability OR “thrombin generation” OR “von Willebrand factor” OR “endothelial activation” OR “D-dimer” OR fibrinogen).

The database search identified 638 records before duplicate removal: PubMed/MEDLINE (*n* = 240), Web of Science Core Collection (*n* = 94), Embase (*n* = 131), and Scopus (*n* = 173). After EndNote-based duplicate removal, 518 records remained for title and abstract screening. Study identification and selection were performed by M.B. and J.H. Disagreements or uncertainties regarding relevance were resolved by discussion, with emphasis on the conceptual focus of the review.

Studies were prioritized if they were directly relevant to radical prostatectomy, perioperative haemostatic changes, postoperative biomarker dynamics, venous thromboembolism, or thromboprophylaxis. Radical prostatectomy-specific clinical and biomarker studies were considered most relevant. When direct radical prostatectomy-specific evidence was limited, selected studies from related urological, oncological, thrombosis, or major surgical settings were included if they provided mechanistic or clinical context relevant to postoperative hypercoagulability, endothelial activation, thrombin generation, von Willebrand factor biology, or post-discharge thrombotic risk. Reviews, guidelines, and key mechanistic articles were included when they contributed directly to the interpretive framework of the manuscript.

Records were excluded if they were duplicates, clearly unrelated to radical prostatectomy or postoperative haemostasis, focused only on unrelated oncological or surgical populations without mechanistic relevance, addressed diagnosis or treatment of established venous thromboembolism without relevance to postoperative risk assessment or prevention, or did not contribute meaningfully to the narrative synthesis. Because this was a narrative review, formal risk-of-bias assessment and study-quality scoring were not performed. However, the included literature was interpreted critically with attention to study design, sample size, biomarker methodology, timing of postoperative assessment, clinical applicability, and the distinction between biological plausibility and clinically validated predictive value.

**Figure 1:**
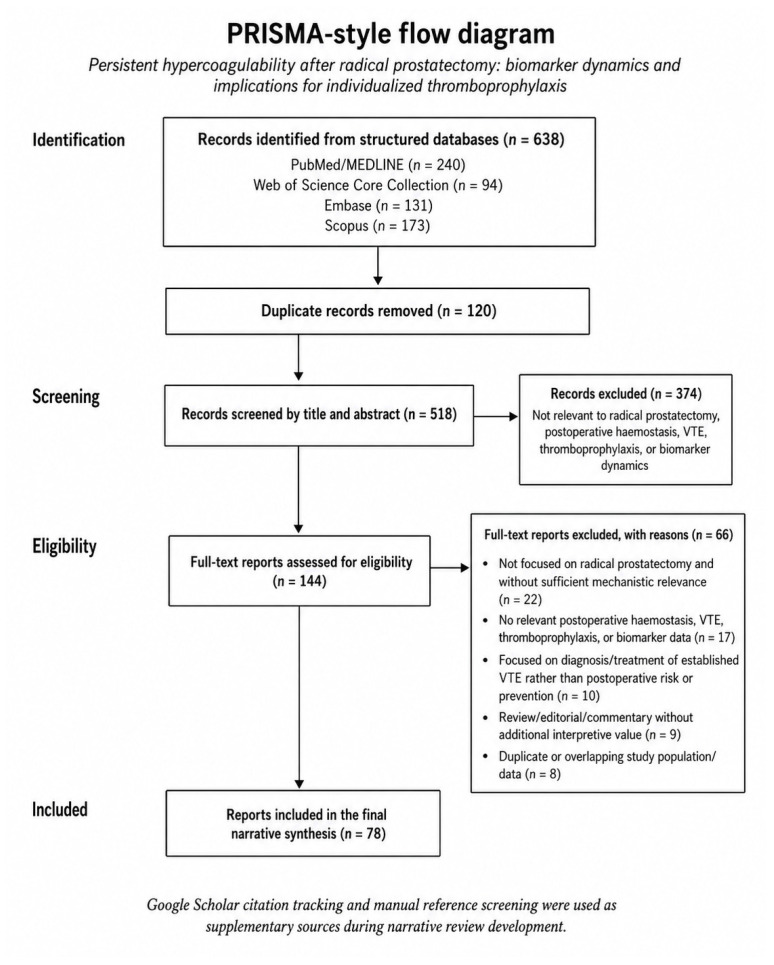
PRISMA-style flow diagram of literature identification, screening, eligibility assessment, and inclusion.

During the revision process, additional relevant reports identified through the updated database search, citation tracking, and reviewer/editorial feedback were incorporated, resulting in 78 reports being included in the final narrative synthesis. A PRISMA-style flow diagram summarizing the process of literature identification, duplicate removal, screening, eligibility assessment, and inclusion is provided in Figure 1.

The figure summarizes the structured database search and study selection process for this focused narrative review. Records were identified in PubMed/MEDLINE, Web of Science Core Collection, Embase, and Scopus, followed by EndNote-based duplicate removal, title/abstract screening, and full-text eligibility assessment. Studies were prioritized according to their relevance to radical prostatectomy, postoperative haemostatic changes, biomarker dynamics, venous thromboembolism, and thromboprophylaxis. Google Scholar citation tracking and manual reference screening were used as supplementary sources.

## 2. Pathophysiological Background

Postoperative hypercoagulability following radical prostatectomy arises from the combined effect of cancer-related, surgery-induced, and endothelial-driven mechanisms [16,17,18]. These processes affect both primary and secondary haemostasis and evolve dynamically over time [17,19]. Furthermore, different components of the haemostatic system may reflect distinct aspects of this response. In this context, biomarkers such as von Willebrand factor and the thrombin generation assay reflect different pathways of haemostasis, thus providing complementary rather than overlapping information on postoperative coagulation changes [12,13,20,21].

### 2.1. Cancer-Associated Hypercoagulability

Cancer is a well-established prothrombotic condition characterized by complex interactions between tumour cells, the coagulation system, and inflammatory responses. Malignant cells can directly activate coagulation through the expression of tissue factor and other procoagulant molecules, while also promoting thrombin generation through the release of procoagulant microparticles. In addition, tumour-associated inflammation contributes to a systemic prothrombotic state by inducing cytokine release, endothelial activation, and platelet aggregation [16,17,18,19].

Together, these mechanisms may lead to a pre-existing imbalance in haemostasis in CaP patients, even before surgical intervention [12,13]. Elevated levels of coagulation-related markers (factor VIII, von Willebrand factor, and increased thrombin-generating potential) have been reported in cancer patients and are associated with an increased risk of venous thromboembolism [20,21]. In contrast, alterations in the von Willebrand factor–ADAMTS13 axis reflect a mechanistic pathway leading to enhanced platelet adhesion and thrombus formation [22].

Together, these mechanisms establish a baseline procoagulant state in CaP patients, which may amplify the haemostatic response to subsequent surgical trauma.

### 2.2. Surgery-Induced Coagulation Activation

Surgical intervention further enhances the prothrombotic state through multiple mechanisms, including direct vascular injury, activation of the coagulation cascade, and systemic inflammatory responses [23]. During RP, surgeons dissect a highly vascularized organ and injure the Santorini venous plexus, leading to endothelial damage and exposure of subendothelial procoagulant surfaces, which in turn initiates a cascade involving platelet activation, thrombin generation, and fibrin formation [12,13].

In addition to local effects, surgery induces a systemic acute-phase response characterized by increased inflammatory mediators, leukocyte activation, and changes in coagulation and fibrinolysis [24]. Elevated levels of fibrinogen, D-dimer, and other acute-phase markers, such as C-reactive protein and factor VIII, have been consistently observed in the early postoperative period, reflecting ongoing coagulation and altered fibrinolytic balance [12,13,25,26].

Importantly, so-called minimally invasive techniques, including laparoscopic and robot-assisted approaches, do not eliminate thrombotic risk, partly because of procedure-specific factors such as pneumoperitoneum and patient positioning [6,12,13,14,27].

Together, cancer-related and surgery-induced mechanisms result in a dynamic and multifactorial prothrombotic state, the temporal evolution of which remains incompletely understood. This underscores the need for integrated assessment of haemostatic changes using complementary biomarkers, as discussed in the following sections.

**Figure 2:**
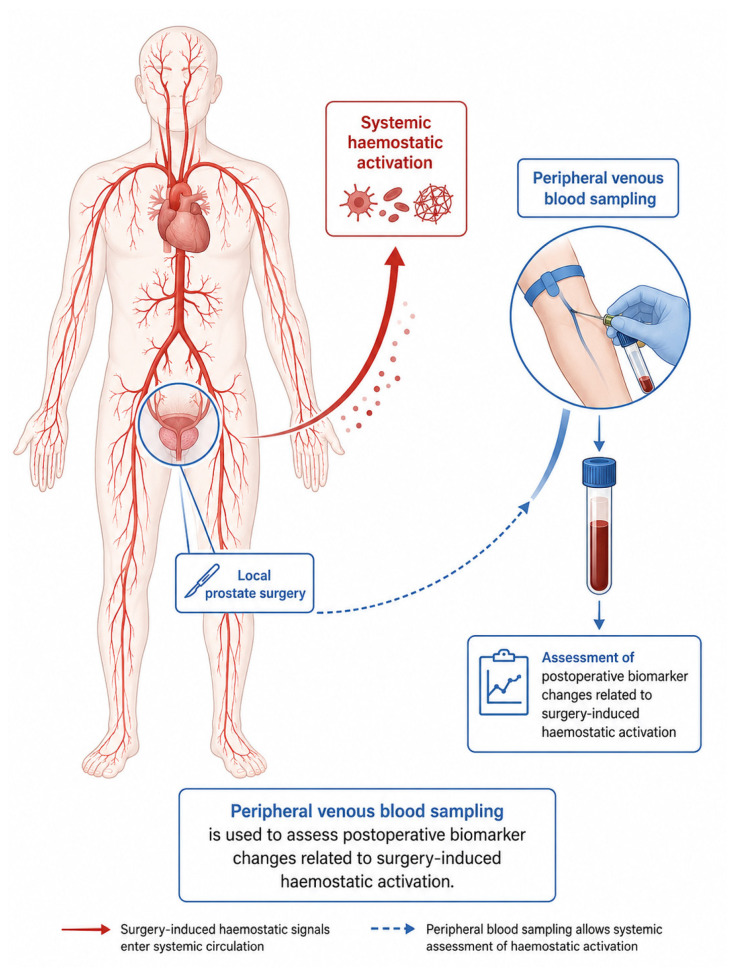
From local prostate surgery to systemic haemostatic activation and peripheral venous biomarker assessment.

As illustrated in Figure 2, local surgical injury may lead to systemic haemostatic activation, which can be assessed through peripheral venous blood sampling.

Figure 2 presents a schematic overview of the proposed relationship between local surgical trauma at the prostate, systemic haemostatic activation, and postoperative biomarker assessment in peripheral venous blood. The figure illustrates that surgery-induced haemostatic signals may enter the systemic circulation and become detectable through postoperative peripheral blood sampling, providing the basis for longitudinal evaluation of biomarker changes after radical prostatectomy.

### 2.3. Endothelial Activation and the Role of Von Willebrand Factor

Endothelial injury is a central component of postoperative hypercoagulability after radical prostatectomy. Tissue damage exposes procoagulant surfaces and promotes the release of mediators such as tissue factor and von Willebrand factor (VWF), thereby initiating platelet recruitment and coagulation activation. In parallel, VWF stabilizes factor VIII and supports thrombin generation, thereby linking primary and secondary haemostasis. An imbalance between endothelial activation, platelet–VWF interaction, coagulation, and fibrinolysis may therefore contribute to venous thrombosis [28,29,30]. Among procoagulant factors, factor VIII and VWF appear to be particularly strongly associated with the risk of venous thrombosis [31].

VWF is synthesized by endothelial cells and megakaryocytes and undergoes complex post-translational modification, including multimerization and glycosylation, which are essential for its structure and function [32]. It circulates as multimers of variable size, and its activity is regulated by shear stress and proteolytic cleavage, particularly by ADAMTS13 [33]. The balance of these processes is critical, as both quantitative and functional alterations in VWF may shift haemostasis toward thrombosis.

Previous studies have described endothelial perturbation and altered VWF and factor VIII levels in prostate cancer, while ADAMTS13 deficiency has also been linked to thrombotic complications in cancer patients [34,35,36,37]. Surgery-related changes in VWF activity and multimer distribution have likewise been reported in other major surgical settings [38,39,40]. However, detailed characterization of primary haemostatic changes after radical prostatectomy remains limited.

In our single-centre longitudinal observational study of 24 prostate cancer patients undergoing radical prostatectomy, with comparison to 24 healthy controls, VWF:Ag and factor VIII levels increased after surgery, whereas the ADAMTS13-related balance decreased. In addition to quantitative elevation, functional changes were also observed, including enhanced collagen-binding activity (VWF:CB) and larger VWF multimers. These alterations were most pronounced at 1 h and 6 days after surgery, respectively, declined toward baseline by 1 month, and decreased further by 10 months, consistent with prolonged endothelial perturbation followed by a gradual return to baseline after curative treatment for prostate cancer. This pattern is consistent with the concept that thrombotic risk may persist at least through the fourth postoperative week [6,12,41,42].

Postoperative leukocytosis returned to baseline by day 6, indicating resolution of the early acute-phase response [43]. By contrast, VWF, factor VIII, fibrinogen, and D-dimer remained altered from baseline for longer, supporting the view that endothelial activation and haemostatic imbalance outlast the immediate inflammatory phase [44,45]. Given the short plasma half-life of VWF, these sustained abnormalities likely reflect not only release from endothelial storage pools but also ongoing endothelial activation and renewed synthesis during vascular repair and re-endothelialization. This interpretation is also consistent with broader evidence that endothelial repair after tissue injury involves coordinated vascular remodelling responses, including VEGF-related signalling pathways that may accompany prolonged endothelial activation during recovery [46]. The associated shift toward more prothrombotic VWF forms and VWF–ADAMTS13 imbalance may represent a biologically relevant component of persistent postoperative hypercoagulability [46,47,48].

Taken together, these findings support the concept that endothelial activation is not merely an early surgical phenomenon but may persist well beyond the immediate postoperative period. Accordingly, VWF-related parameters may prove useful as markers of prolonged haemostatic perturbation and warrant further evaluation in relation to postoperative thrombotic risk after radical prostatectomy.

### 2.4. Global Coagulation Changes: Thrombin Generation Assay

Hypercoagulability reflects a shift in the coagulation system toward a prothrombotic state, even in the absence of clinically manifest thrombosis. Conventional coagulation tests, including prothrombin time, activated partial thromboplastin time, thrombin time, fibrinogen, and D-dimer, are routinely used to evaluate haemostasis, but they provide only limited information on the global dynamics of coagulation activation [49].

The thrombin generation assay (TGA) offers a more integrative assessment of haemostatic potential by measuring thrombin formation in clotting plasma ex vivo [50]. After recalcification of citrated plasma, coagulation is triggered by tissue factor and phospholipids, allowing quantification of the kinetics and magnitude of thrombin generation [51]. Under appropriate pre-analytical and analytical conditions, the TGA provides a dynamic measure of overall coagulation capacity and may be useful for assessing thrombotic risk and treatment effects [49,52,53]. Elevated basal thrombin generation has been associated with an increased risk of venous thromboembolism in prospective studies [52,54].

TGA parameters are influenced by multiple biological determinants, including tissue factor activity and circulating microparticles [51,55]. In our prospective, single-centre, longitudinal study, 24 patients undergoing radical prostatectomy were compared with 20 age-matched healthy controls; preoperative TGA parameters were already higher in the patient group, consistent with baseline cancer-associated procoagulant activity. Radical prostatectomy further amplified this response, with postoperative increases in thrombin-generating potential that gradually declined by the end of the first month. Differences compared with the healthy controls only disappeared after 10 months, indicating that both malignancy-related and surgery-induced mechanisms shape the temporal profile of thrombin generation [12,20]. Blood samples were collected at D−1, H1, D6, M1, and M10, allowing longitudinal assessment of postoperative thrombin-generating capacity. In contrast to the TGA, the conventional clotting times remained largely within reference ranges, and the platelet counts showed only limited variation. Correlation analyses suggested that increased procoagulant activity was more readily captured by the TGA than by routine coagulation tests. D-dimer elevation was consistent with previous observations, whereas fibrinogen showed a slower postoperative response than D-dimer and peak thrombin generation [56]. We did not observe a correlation between fibrinogen and peak thrombin generation, suggesting that fibrinogen contributed less directly to the increase in thrombin-generating capacity in this setting.

Overall, these findings suggest that the TGA may be particularly useful for tracking subtle but clinically relevant changes in global coagulation potential after radical prostatectomy; however, broader clinical implementation remains limited by pre-analytical variability and incomplete assay standardization across laboratories [50,52].

## 3. Dynamics of Postoperative Hypercoagulability

Postoperative hypercoagulability following radical prostatectomy is not a static phenomenon but a time-dependent process characterized by distinct phases of haemostatic activation and recovery [1,2,3,4]. Comparison of preoperative and postoperative markers demonstrates that different components of haemostasis follow divergent temporal patterns, reflecting the combined effects of surgical trauma, inflammation, and endothelial activation [7,13,19].

Importantly, the return of haemostatic markers to baseline levels does not occur simultaneously, with some taking weeks and others remaining altered for a prolonged period, suggesting that biological recovery of the haemostatic system may extend beyond the timeframe typically covered by standard thromboprophylaxis [12,13,57].

This temporal dissociation raises the possibility of a clinically relevant post-discharge risk period, during which conventional risk assessment may underestimate thrombotic risk [8,11,12,58,59].

**Figure 3:**
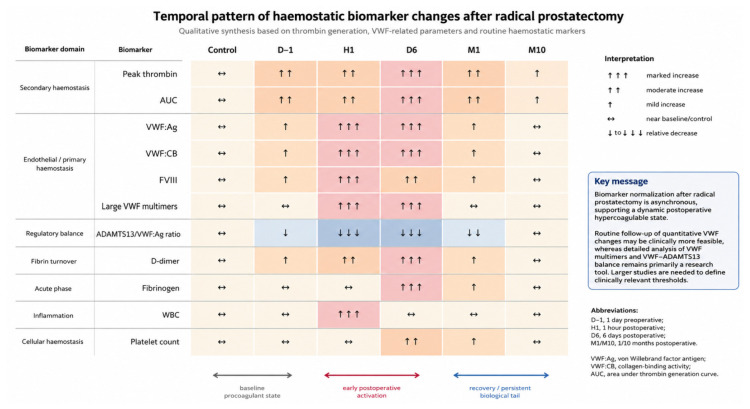
Temporal pattern of haemostatic biomarker changes after radical prostatectomy. Abbreviations: D−1, 1 day pre-surgery; H1, 1 h post-surgery; D6, 6 days post-surgery; M1, 1 month post-surgery; M10, 10 months post-surgery; VWF:Ag, von Willebrand factor antigen; VWF:CB, collagen-binding activity; AUC, area under the thrombin generation curve.

Figure 3 summarizes the temporal pattern of postoperative biomarker changes after radical prostatectomy and illustrates that the return of haemostatic parameters to baseline is asynchronous across different pathways.

Qualitative summary of perioperative changes in thrombin generation, von Willebrand factor (VWF)-related parameters, and routine haemostatic markers across the postoperative course. The figure illustrates asynchronous return to baseline of biomarkers after radical prostatectomy, supporting a dynamic postoperative hypercoagulable state. Changes in thrombin generation and VWF-related parameters suggest that biological recovery may extend beyond the early postoperative phase. Routine follow-up of quantitative VWF changes may be the most clinically feasible VWF-related approach in practice, provided that results are interpreted within appropriate reference ranges. In contrast, detailed assessment of VWF multimer distribution and the VWF–ADAMTS13 regulatory balance remains primarily a research laboratory tool at present. Larger studies are needed to validate these findings and define clinically relevant biomarker thresholds.

### 3.1. Early Postoperative Period

The early postoperative period following radical prostatectomy is characterized by a well-documented hypercoagulable state [2,3,4]. Surgical trauma leads to immediate activation of the coagulation system, reflected by increased thrombin generation, elevated levels of coagulation-related markers, and enhanced platelet activation [12,13].

Within hours after surgery, von Willebrand factor levels and activity increase, accompanied by the appearance of larger multimers and a relative decrease in ADAMTS13 activity [13,60]. These changes indicate acute endothelial activation and contribute to enhanced platelet adhesion and aggregation. In parallel, thrombin-generating potential is increased, reflecting activation of secondary haemostasis [12].

In the first postoperative days, additional changes become evident, including elevated D-dimer levels, increased fibrinogen, and persistent inflammatory activation [12,13,45]. These findings indicate ongoing coagulation and fibrinolysis and support the concept of an early postoperative prothrombotic state.

Although these alterations are expected and partly addressed by standard thromboprophylaxis, they indicate that the haemostatic system remains markedly activated in the early postoperative phase. Importantly, the magnitude and persistence of these changes may vary between patients and are not fully captured by routine clinical risk assessment models [6,7].

### 3.2. Beyond Hospital Discharge

Although the immediate postoperative phase is marked by clear haemostatic activation, biological recovery does not necessarily keep pace with clinical discharge. After radical prostatectomy, several haemostatic abnormalities persist beyond the first postoperative days, indicating that discharge from hospital does not necessarily reflect normalization of the coagulation system or recovery of all haemostatic pathways [12,13].

Our follow-up studies suggested that the postoperative trajectories of thrombin generation and endothelial biomarkers are not identical. Thrombin generation parameters remained elevated during the first postoperative month and approached control values only later during follow-up, consistent with a sustained increase in global coagulation potential [12]. Similarly, von Willebrand factor-related parameters, including antigen levels, collagen-binding activity, multimer distribution, and ADAMTS13-related changes, showed persistent postoperative alterations that extended beyond the early inflammatory phase [13]. Together, these findings indicate that both secondary haemostasis and endothelial activation may remain disturbed after hospital discharge. However, these observations are based on a limited number of small single-centre longitudinal studies and should therefore be interpreted as biomarker-based evidence of persistent biological activation rather than direct proof of clinically overt thrombotic risk.

This delayed return to baseline is also consistent with observations from other haemostatic markers. Postoperative D-dimer levels may remain elevated for several weeks after major surgery, reflecting ongoing activation of coagulation and fibrinolysis rather than a short-lived perioperative response [45,57]. In parallel, clinical studies have shown that a substantial proportion of postoperative venous thromboembolic events occur after discharge, with risk persisting throughout the first postoperative month [12,56,57]. More recent pooled evidence supports this temporal pattern, demonstrating that symptomatic postoperative VTE events are distributed across the first 4 weeks after surgery rather than being confined to the immediate postoperative period [61]. Taken together, these data support temporal and biological plausibility, but they do not establish a direct biomarker-defined post-discharge risk window at the individual-patient level.

Overall, postoperative haemostatic recovery after radical prostatectomy appears to be prolonged and biologically heterogeneous. Persistence of abnormal thrombin generation, endothelial perturbation, and fibrinolytic activity beyond hospital discharge provides a plausible mechanistic explanation for a biologically relevant post-discharge phase, although the direct relationship to clinically manifest VTE remains insufficiently established. Prospective studies are needed to determine whether these late haemostatic alterations can support individualized post-discharge risk stratification before they can be considered clinically actionable [12,13].

### 3.3. Biomarkers: Their Kinetics and Clinical Interpretation

The presence of biomarkers can help characterize postoperative hypercoagulability, but the specific meaning depends strongly on both biological context and timing. Their value in this setting is currently interpretive and hypothesis-generating rather than directly decision-guiding. There is no single marker that fully reflects the complexity of postoperative haemostatic changes following radical prostatectomy. Instead, different biomarkers reflect different components of the prothrombotic response, including endothelial activation, thrombin-generating capacity, fibrinolytic activity, and systemic inflammation [12,13,45,60].

In this context, parameters related to the thrombin generation assay and von Willebrand factor are of particular interest because they provide complementary rather than overlapping information: Thrombin generation reflects the global capacity of plasma to generate thrombin and therefore serves as an integrative marker of secondary haemostasis [12,49,52]. In contrast, von Willebrand factor, together with ADAMTS13-related changes and multimer distribution, reflects endothelial perturbation and platelet-adhesive potential, thereby capturing an important aspect of primary haemostasis [13,33,47,60]. This distinction matters clinically, because postoperative hypercoagulability after radical prostatectomy seems to involve both sustained coagulation activation and prolonged endothelial dysfunction. This does not imply that TGA- and VWF-related markers are already clinically superior predictors of postoperative VTE; rather, their potential value lies in the fact that they capture complementary haemostatic domains and may therefore be particularly informative for longitudinal biological assessment.

The temporal behaviour of these markers is equally important. Our follow-up studies showed early postoperative increases in thrombin-generating potential and von Willebrand factor levels and activity, larger multimers, reduced ADAMTS13 activity, and elevated D-dimer levels, with incomplete normalization during the first postoperative month [12,13]. These findings suggest that haemostatic recovery is not synchronous across different pathways. Some abnormalities improve relatively early, whereas others persist for weeks or even months, indicating that postoperative recovery should be viewed as a dynamic process rather than a single postoperative state. However, these kinetic patterns should be interpreted primarily as evidence of biological heterogeneity in haemostatic recovery, rather than as validated markers of individual clinical thrombotic risk.

Other markers, such as D-dimer, fibrinogen, C-reactive protein, and leukocyte-related inflammatory indices, provide additional but less specific information. D-dimer reflects ongoing coagulation and fibrinolysis and may remain elevated for several weeks after surgery [45,57]. Similarly, inflammatory markers may remain abnormal during the postoperative period, but their interpretation is limited by poor specificity, as they may reflect tissue injury, inflammation, infection, or malignancy-related processes rather than thrombotic risk alone [62,63]. Therefore, although such markers may support the presence of a persistent biological response, they are less suitable as isolated tools for individualized risk prediction. This broader lack of specificity across postoperative biomarkers underscores the difficulty in translating biological activation into clinically actionable risk stratification.

A central challenge is that abnormal biomarker levels do not necessarily translate into clinical thrombotic events. Biological plausibility and statistical association are not equivalent to clinical utility. As discussed in later sections, many candidate biomarkers show inconsistent predictive performance when evaluated as standalone markers, particularly in heterogeneous perioperative populations [64,65,66,67,68]. This limitation is especially relevant in radical prostatectomy, where clinically manifest VTE is relatively infrequent, while subclinical or biological evidence of hypercoagulability is much more common. Similar caution should be applied in the case of TGA- and VWF-related parameters: despite their biological relevance, their predictive performance for clinically manifest postoperative VTE after radical prostatectomy needs to be prospectively validated.

For this reason, the greatest value of postoperative biomarkers may lie not in single-threshold prediction but in longitudinal assessment and integrated interpretation. Persistent elevation of thrombin generation and D-dimer, as well as von Willebrand factor-related abnormalities, beyond the immediate postoperative phase may indicate delayed haemostatic recovery and support the concept of a biologically relevant post-discharge risk period [12,13,45,61]. However, clinically actionable thresholds remain undefined. At present, this interpretation remains conceptual and should not be viewed as equivalent to a validated risk prediction model.

Thus, current evidence suggests that the main clinical relevance of postoperative biomarkers after radical prostatectomy lies in their ability to characterize the kinetics and heterogeneity of haemostatic recovery. Their role is therefore not to replace clinical decision-making at present, but to improve understanding of the temporal mismatch between biological recovery and fixed-duration thromboprophylaxis. Future studies should determine whether combinations of complementary biomarkers, assessed longitudinally rather than at a single time point, provide clinically useful information for postoperative risk stratification beyond biological characterization alone.

The main postoperative haemostatic biomarkers, and their biological interpretation, temporal behaviour, and potential clinical relevance, are summarized in Table 1.

The table summarizes the principal haemostatic biomarkers discussed in this review, with emphasis on the biological pathway they reflect, their typical postoperative behaviour after radical prostatectomy, their potential clinical relevance, and their main limitations. The listed patterns are intended as qualitative summaries of the currently available evidence rather than validated thresholds for clinical decision-making.

### 3.4. Evolution of Surgical Techniques and Thrombotic Risk

Advances in surgical techniques have substantially improved perioperative outcomes in urological surgery, including reduced blood loss, shorter hospital stay, and faster recovery [69,70]. So-called minimally invasive approaches, particularly laparoscopic and robot-assisted radical prostatectomy, have become widely adopted and are often associated with lower perioperative morbidity [5]. Contemporary radical prostatectomy studies have additionally emphasized individualized surgical planning based on preoperative imaging, tumour characteristics, and patient-specific risk assessment to optimize both oncological and functional outcomes [71].

However, these advances have not eliminated thrombotic risk. Recent systematic review evidence specific to radical prostatectomy also suggests that overall postoperative VTE incidence is relatively low, but not negligible, and may vary according to surgical approach and the performance of pelvic lymph node dissection [72]. The relationship between surgical technique and venous thromboembolism remains complex, and available data are partly conflicting. While shorter hospitalization and earlier mobilization may reduce thrombotic risk, procedure-specific factors may counteract these benefits. Although minimally invasive approaches are associated with smaller incisions and more precise dissection, the extent of internal surgical trauma may remain comparable to open procedures, as similar anatomical structures are exposed and dissected, possibly contributing to a sustained prothrombotic response [7,12,13,69,70].

Laparoscopic and robot-assisted procedures require pneumoperitoneum and steep Trendelenburg positioning, which may impair venous return and promote venous stasis. In addition, increased intra-abdominal pressure and ventilatory adjustments during surgery may further influence haemodynamic and coagulation-related factors. These may contribute to a prothrombotic environment despite less invasive surgical access [27,71].

Importantly, the current evidence does not consistently demonstrate a significantly lower incidence of venous thromboembolism with minimally invasive techniques compared to open surgery [70,71,72,73,74]. This suggests that improvements in surgical precision and perioperative care do not necessarily translate into a proportional reduction in thrombotic risk.

So, advances in surgical techniques have not eliminated thrombotic risk and may introduce new pathophysiological factors, highlighting the need to consider procedure-specific mechanisms when assessing postoperative thrombotic risk and suggesting that surgical technique alone is insufficient for thrombotic risk stratification.

### 3.5. Emerging Biomarkers and Their Clinical Limitations

A growing number of biomarkers have been investigated to improve the prediction and diagnosis of venous thromboembolism. However, despite promising mechanistic insights, their clinical applicability in perioperative settings remains limited.

Markers reflecting endothelial activation and inflammation, such as soluble P-selectin, thrombomodulin, and neutrophil extracellular traps (NETs), have been associated with thrombotic processes in various clinical contexts [64,65,66]. In selected populations, including patients with malignancy receiving chemotherapy, elevated levels of P-selectin and NET-related markers have shown potential predictive value for thrombotic events [65]. Similarly, thrombomodulin has been proposed as a dynamic biomarker reflecting endothelial injury and thrombus burden [66].

However, these findings are not consistent across studies. Large population-based analyses have failed to demonstrate a robust association between elevated P-selectin levels and incident venous thromboembolism in the general population. In a recent case–control study including over 400 VTE events, soluble P-selectin was not independently associated with future VTE risk overall, although subgroup-specific associations were observed [64]. These discrepancies highlight the context-dependent performance of individual biomarkers.

Evidence from perioperative and trauma-related settings is similarly heterogeneous. In prospective surgical cohorts, biomarkers such as P-selectin, tissue factor, and collagen-related markers did not show a consistent association with postoperative deep vein thrombosis, despite their established pathophysiological role in coagulation activation [45]. These findings suggest that biological relevance does not necessarily translate into clinical predictive value.

Early pilot studies evaluating combinations of biomarkers have demonstrated modest improvements in diagnostic performance. A study assessing D-dimer, soluble P-selectin, and circulating microparticles reported improved sensitivity and specificity when markers were combined; however, overall diagnostic accuracy remained moderate, at approximately 70–80% [67]. This level of performance is insufficient to replace or reliably complement established diagnostic strategies.

More recent approaches have explored the integration of clinical risk models with biomarker data. For example, combining the Caprini score with molecular markers has been reported to improve predictive accuracy in surgical patients; however, these models remain limited by heterogeneity in study populations and a lack of external validation. Similarly, machine learning-based risk stratification models incorporating biomarker data have shown promising results, but their clinical applicability remains uncertain and requires prospective validation [68].

In addition, inflammatory biomarkers such as C-reactive protein, the neutrophil-to-lymphocyte ratio, and other acute-phase parameters have been associated with increased thrombotic risk in large cohorts; however, their lack of specificity limits their utility for individualized risk prediction [62,63].

Importantly, most available studies are characterized by relatively small sample sizes, short follow-up periods, and heterogeneous patient populations. Furthermore, many biomarkers are assessed at single time points, which does not reflect the dynamic nature of postoperative haemostatic changes.

Thus, current evidence suggests that the main limitation of emerging biomarkers is not their biological plausibility but their insufficient and inconsistent clinical performance. This translational gap is not unique to thrombotic biomarkers; biologically promising associations in other radical prostatectomy-related biomarkers have also proven difficult to translate into clinically actionable risk stratification tools [75]. Rather than relying on individual markers, future strategies may benefit from combining complementary biomarkers that reflect different components of haemostasis together with longitudinal assessment of their temporal dynamics.

## 4. Clinical Implications

The following section focuses on the clinical implications of the pathophysiology and temporal dynamics of postoperative hypercoagulability for risk assessment and thromboprophylaxis.

### 4.1. Limitations of Current Risk Assessment Models

Current risk assessment models for venous thromboembolism (VTE) in surgical patients are largely based on static variables, including patient characteristics, comorbidities, and procedural factors [8,9,10]. While these models provide a practical framework for perioperative risk stratification, they do not account for the dynamic and time-dependent nature of postoperative haemostatic changes [10].

In particular, these models fail to incorporate temporal variations in coagulation and endothelial function, which may significantly influence thrombotic risk during the postoperative period [12,13]. As a consequence, risk assessment is typically performed at a single time point, without considering the evolution of haemostatic alterations after surgery.

Furthermore, most currently used models do not integrate biomarker data, despite increasing evidence that markers such as thrombin generation and von Willebrand factor reflect complementary and clinically relevant aspects of postoperative hypercoagulability [12,13,60]. This limitation reduces their ability to capture interindividual variability in thrombotic risk.

Thus, current risk assessment approaches may be insufficient to fully characterize postoperative thrombotic risk, particularly in the context of modern surgical techniques, evolving perioperative care, and time-dependent haemostatic recovery [9,10].

### 4.2. Post-Discharge Thrombotic Vulnerability: Biological Plausibility and Clinical Uncertainty

The concept of a post-discharge risk period after radical prostatectomy arises from the temporal mismatch between clinical recovery and biological recovery of the haemostatic system. At present, however, this concept is supported mainly by biomarker persistence and the timing of postoperative events, rather than by direct prospective validation of biomarker-defined clinical risk. This concept is relevant because discharge from hospital does not necessarily coincide with return to baseline of haemostatic pathways. Clinical cohorts and biomarker studies both suggest that postoperative thrombotic vulnerability may extend beyond the early period traditionally covered by standard thromboprophylaxis, with large population-based and registry studies consistently demonstrating that a substantial proportion of postoperative venous thromboembolism (VTE) events occur after hospital discharge. In a landmark analysis, the median time to VTE diagnosis after surgery was approximately 16 days, with a substantial proportion of events occurring beyond the first postoperative week [59]. Similarly, prospective cohort data indicate that up to 40–60% of postoperative VTE events develop after discharge, with risk persisting for several weeks [11,58]. A recent meta-analysis further strengthened this concept by showing that symptomatic postoperative VTE events are distributed across the entire first postoperative month, with 47.1% occurring during the first week after surgery, 26.9% during the second, 15.8% during the third, and 10.1% during the fourth [61]. These data support the temporal persistence of postoperative thrombotic risk, but in isolation do not define the biological mechanisms underlying individual post-discharge vulnerability.

These clinical observations are biologically consistent with laboratory evidence indicating prolonged postoperative activation of haemostasis. Postoperative D-dimer levels typically peak around postoperative-day 5–7 and may remain elevated for several weeks, reflecting sustained activation of coagulation and fibrinolysis [57]. In parallel, inflammatory markers, such as C-reactive protein, may remain elevated, indicating a persistent systemic inflammatory response [63]. However, these laboratory abnormalities remain non-specific and should not be interpreted as direct surrogate markers of clinically manifest postoperative VTE.

Importantly, the limited biomarker studies in radical prostatectomy patients provide a biologically plausible basis for this concept. Persistent increases in thrombin-generating potential and sustained alterations in von Willebrand factor parameters have been observed well beyond the immediate postoperative phase [12,13]. These findings are consistent with delayed return to baseline of the haemostatic balance, but they do not establish a direct predictive relationship between biomarker persistence and clinically manifest post-discharge VTE.

Taken together, these findings support the concept of a biologically relevant post-discharge phase during which thrombotic vulnerability may persist despite apparent clinical recovery. Such a phase is not adequately captured by conventional perioperative risk assessment and may partly explain why fixed-duration strategies do not always align with postoperative biological recovery. However, the direct relationship between persistent biomarker abnormalities and clinically manifest post-discharge VTE remains insufficiently established, and the concept should therefore be regarded as biologically plausible rather than clinically validated.

### 4.3. Extended Thromboprophylaxis: Evidence and Controversies

The optimal duration of postoperative thromboprophylaxis remains a subject of ongoing debate. Current guidelines generally suggest extended prophylaxis for approximately four weeks after major surgery; however, these recommendations are largely extrapolated from non-urological surgical populations and supported by limited procedure-specific evidence [8,10,72]. A more individualized perspective is reflected in recent European urological guidance, which supports a tailored approach based primarily on procedural and patient-related factors, although biomarker dynamics have not yet been incorporated into the recommendations [8].

A central principle underlying these recommendations is the balance between thrombotic and bleeding risks. Pharmacological thromboprophylaxis reduces the relative risk of VTE by approximately 40–60% but is associated with an increased risk of bleeding complications [10]. Consequently, guidelines reflect a population-level trade-off rather than an individualized assessment of risk. As a result, the net clinical benefit of extended prophylaxis is unlikely to be uniform across all patients undergoing radical prostatectomy.

Importantly, the temporal distribution of postoperative complications is asymmetric. Recent pooled evidence shows that more than half of symptomatic postoperative VTE events occur after the first postoperative week, whereas bleeding events are concentrated earlier in the postoperative course [10,61,73]. This pattern provides a rationale for considering extended thromboprophylaxis in selected patients, but it does not by itself define the optimal duration of prophylaxis for an individual patient. Recent retrospective robotic prostatectomy data also support a risk-adapted approach, as routine extended pharmacologic prophylaxis was associated with higher bleeding morbidity without clear evidence of thrombotic benefit [76].

The limited available biomarker studies suggest that haemostatic activation may persist beyond the standard prophylaxis period. Sustained elevation of thrombin generation and endothelial activation markers suggests that biological recovery may lag behind clinical recovery [12,13]. This raises the question of whether fixed-duration prophylaxis adequately reflects the temporal dynamics of postoperative hypercoagulability. At the same time, persistent biomarker abnormalities should not be interpreted as sufficient evidence that prophylaxis ought to be extended in individual patients without prospective clinical validation.

Evidence supporting further extension of thromboprophylaxis remains limited, particularly in urological surgery. Randomized controlled trials specifically addressing extended prophylaxis in this population are lacking, and the available data are heterogeneous [72]. In addition, prolonged anticoagulation increases bleeding risk, underscoring the need for careful patient selection and balanced assessment of net clinical benefit. In the absence of validated biomarker thresholds or prospective biomarker-guided trials, extending anticoagulation on the basis of persistent biomarker abnormalities alone may expose some patients to additional bleeding without proven individual benefit.

In conclusion, current thromboprophylaxis strategies represent a pragmatic compromise rather than a biologically tailored approach. The discrepancy between fixed-duration recommendations and dynamic haemostatic changes highlights a clinically relevant gap and suggests that future strategies may benefit from individualized, time-dependent risk assessment and provides a rationale for exploring biomarker-informed thromboprophylaxis strategies in future prospective studies.

### 4.4. Are Current Risk Models Outdated?

Current risk assessment models for venous thromboembolism in surgical patients are primarily based on static clinical variables, including patient characteristics, comorbidities, and procedural factors [10]. Current urological guidance already acknowledges that net benefit may vary substantially according to procedure type, surgical approach, extent of lymphadenectomy, and patient-level thrombotic risk, highlighting the limitations of static one-size-fits-all models [8]. While these models provide a practical framework for perioperative decision-making, they do not reflect the dynamic nature of postoperative haemostatic changes. Baseline procedural risks of thrombosis and bleeding are themselves difficult to estimate reliably, which further complicates the development of robust procedure-specific risk models [71]. Although procedure-specific VTE prediction models have been proposed for robot-assisted radical prostatectomy, they remain insufficiently validated and do not incorporate postoperative biomarker dynamics [77].

Importantly, contemporary surgical practice has evolved substantially, with widespread adoption of minimally invasive and robot-assisted techniques. However, although these approaches improve perioperative outcomes, they introduce procedure-specific factors—such as pneumoperitoneum, patient positioning, and altered haemodynamics—that are not adequately represented in existing risk models [73].

Furthermore, current models do not incorporate biomarker data, despite growing evidence that markers of endothelial activation and global coagulation provide clinically relevant information on postoperative hypercoagulability [12,13]. In addition, they fail to account for the temporal evolution of haemostatic alterations, which may extend beyond the early postoperative period.

Taken together, these limitations suggest that current risk models may underestimate thrombotic risk, particularly in the post-discharge phase. This gap highlights the potential value of future risk stratification approaches that may eventually integrate clinical variables with biomarker dynamics and time-dependent haemostatic changes.

## 5. Prevention and Thromboprophylaxis: Towards a Biomarker-Informed Approach

Recent large-scale real-world data from major urologic cancer surgery also support the need for risk-stratified thromboprophylaxis, as clinically relevant VTE persists despite high adherence to current prophylactic protocols [78].

Even with clear advances in surgical technique, thrombotic risk after radical prostatectomy has not disappeared, suggesting that procedural factors alone cannot fully explain postoperative hypercoagulability [8,73]. Current thromboprophylaxis strategies are largely based on guideline recommendations that apply fixed-duration approaches according to population-level risk stratification. While this strategy is practical and has improved perioperative safety, it does not account for interindividual variability in postoperative haemostatic response and recovery.

Importantly, the available evidence suggests that while not all patients require extended thromboprophylaxis, a subset may remain at increased thrombotic risk beyond the standard prophylaxis period [10,12]. This highlights the need for a more individualized approach to postoperative prevention. At present, however, such patients cannot be identified reliably in routine practice on the basis of validated biomarker-guided criteria.

A key limitation of current practice is the lack of integration of dynamic haemostatic information. Biomarker studies have shown that markers reflecting different components of haemostasis—such as thrombin generation and endothelial activation—follow distinct temporal patterns after surgery [12,13]. These findings suggest that postoperative thrombotic risk is not uniform over time and may not be adequately captured by fixed-duration strategies. However, while mechanistically informative, these observations do not establish how biomarker persistence should be translated into individualized prophylaxis decisions.

In this context, a biomarker-informed approach to thromboprophylaxis represents a rational direction for future research. Notably, recent urological guidance has already moved toward more individualized thromboprophylaxis decisions based on procedural and patient-specific risk, although biomarker dynamics are not yet incorporated into this framework [8]. Future studies may identify biologically distinct recovery patterns with different prophylactic needs and should determine whether patients with more persistent postoperative haemostatic activation derive net benefit from longer prophylaxis while those with faster biological recovery do not.

However, such an approach remains hypothetical and requires prospective validation. Currently, there is insufficient evidence to support routine biomarker-guided decision-making, and the balance between thrombotic risk reduction and bleeding complications must remain a central consideration [8,10]. In particular, extending anticoagulation on the basis of persistent biomarker abnormalities alone could expose some patients to additional bleeding without proven benefit.

Taken together, these considerations suggest that future thromboprophylaxis strategies may benefit from more individualized models that integrate clinical risk factors with the temporal dynamics of haemostatic alterations. Future research should focus on validating biomarker-based models and defining clinically relevant thresholds to guide personalized thromboprophylaxis.

A conceptual framework may be proposed in which postoperative thromboprophylaxis is considered in relation to biomarker kinetics. Future studies should determine whether early postoperative biomarker assessment can identify subgroups with delayed haemostatic recovery who might derive net benefit from longer prophylaxis. At present, however, this framework remains hypothesis-generating and should not be interpreted as a validated basis for individualized clinical decision-making.

## 6. Limitations of the Current Evidence

The available evidence on persistent postoperative hypercoagulability after radical prostatectomy remains subject to several important limitations. First, much of the literature reports relatively small studies, often with a single-centre design and heterogeneous patient populations, especially for biomarker studies, in which sample size, timing of blood sampling, assay methodology, and follow-up duration vary substantially. Such variability limits direct comparison between studies and reduces the strength of conclusions regarding the magnitude and duration of postoperative haemostatic alterations. A major limitation of the available literature is that direct radical prostatectomy-specific biomarker evidence remains scarce and is derived largely from small single-centre longitudinal studies, which limits generalizability and reinforces the exploratory nature of current biomarker-based concepts. In addition, the absence of validated biomarker thresholds and prospective biomarker-guided trials limits not only thrombotic risk stratification but also reliable estimation of net clinical benefit, particularly in relation to the competing risk of postoperative bleeding.

Second, although radical prostatectomy is the central focus of this review, part of the current evidence base is necessarily extrapolated from broader surgical, oncological, or thrombotic settings. This is especially true for studies evaluating extended thromboprophylaxis, emerging biomarkers, and postoperative VTE timing. While such data provide important mechanistic and clinical context, they do not fully replace procedure-specific evidence derived from patients undergoing radical prostatectomy.

Third, a major limitation is the mismatch between biological and clinical endpoints. Persistent abnormalities in thrombin generation, von Willebrand factor-related parameters, D-dimer, and other markers support the concept of prolonged postoperative haemostatic activation, but the direct relationship between these biomarker changes and clinically manifest venous thromboembolism remains insufficiently established. This is particularly important in radical prostatectomy, where overt VTE events are relatively infrequent, whereas biological evidence of hypercoagulability is considerably more common.

Finally, clinically actionable biomarker thresholds have not yet been defined, and no prospective trials have validated biomarker-guided thromboprophylaxis strategies in this setting. Therefore, although the available evidence supports the concept of a prolonged and biologically heterogeneous postoperative prothrombotic state, its translation into individualized clinical decision-making remains preliminary.

## 7. Future Directions

Future research should define more precisely the temporal profile and clinical significance of postoperative hypercoagulability after radical prostatectomy. In particular, larger prospective studies are needed to determine whether persistent biomarker abnormalities are associated with clinically overt venous thromboembolic events and to validate the concept of a biologically relevant post-discharge risk period.

Another priority will be the standardization of biomarker assessment and the integration of biomarker data with clinical and procedural risk factors. Longitudinal studies using harmonized sampling time points and a reproducible laboratory methodology will be essential to define clinically relevant thresholds and to evaluate whether biomarker-informed thromboprophylaxis can improve individualized postoperative management. Until such data are available, biomarker-guided strategies should be regarded as investigational.

## 8. Conclusions

Postoperative hypercoagulability after radical prostatectomy should be viewed as a dynamic and biologically heterogeneous process rather than a transient perioperative event. Evidence from biomarker studies and clinical observations suggests that haemostatic recovery may extend beyond the early postoperative phase and may not be adequately reflected by current fixed-duration thromboprophylaxis strategies.

Persistent abnormalities in thrombin generation, von Willebrand factor-related parameters, and other haemostatic markers support the biological plausibility of a post-discharge phase of persistent haemostatic activation. At the same time, current risk models remain largely static and do not incorporate the temporal evolution of postoperative haemostatic changes.

Overall, radical prostatectomy may serve as a clinically relevant model for studying the relationship between postoperative biomarker dynamics and thrombotic vulnerability. The implications of these findings for individualized thromboprophylaxis remain to be established.

## Figures and Tables

**Table 1:** Overview of haemostatic biomarkers after radical prostatectomy: biological meaning, postoperative pattern, potential clinical relevance, and main limitations.

Biomarker/Parameter	Haemostatic Domain	Typical Postoperative Pattern	Potential Clinical Relevance	Main Limitations
Thrombin generation (peak thrombin, AUC)	Secondary haemostasis/global coagulation potential	Elevated preoperatively compared with healthy controls; further postoperative increase; incomplete normalization during the first postoperative month	Integrative marker of global procoagulant potential; may help identify delayed haemostatic recovery	Limited assay standardization; pre-analytical variability; no validated thresholds for routine postoperative risk stratification
VWF:Ag	Endothelial activation/primary haemostasis	Marked early postoperative increase; partial decline over time; may remain abnormal beyond the immediate inflammatory phase	Quantitative and clinically feasible marker of persistent endothelial activation	Influenced by inflammation and other systemic factors; no established postoperative cut-offs
VWF:CB	Functional VWF activity/platelet-adhesive potential	Early postoperative increase, broadly paralleling VWF:Ag, with evidence of enhanced functional activity	Reflects qualitative as well as quantitative VWF-related changes	Less widely available than quantitative VWF assays; clinical interpretation remains uncertain
Large VWF multimers and VWF–ADAMTS13 balance	Regulatory balance of primary haemostasis	Early increase in larger multimers with relative reduction in ADAMTS13-related balance	May reflect a more prothrombotic endothelial phenotype and prolonged disturbance of primary haemostasis	Primarily research-based assessment; not suitable for routine clinical use at present
Factor VIII	Link between endothelial activation and coagulation	Early postoperative increase, often paralleling VWF-related changes	Associated with risk of venous thrombosis; may support interpretation of endothelial activation	Limited specificity; difficult to interpret in isolation
D-dimer	Fibrin turnover/coagulation and fibrinolysis	Early postoperative increase; may remain elevated for several weeks	Supports the concept of persistent postoperative haemostatic activation and fibrinolytic response	Poor specificity; elevation may reflect surgery, inflammation, tissue injury, or thrombosis
Fibrinogen	Acute-phase response/clot substrate	Slower postoperative increase than D-dimer or thrombin generation; may remain elevated during recovery	Supports interpretation of ongoing postoperative prothrombotic and inflammatory response	Acute-phase reactant with low specificity for thrombotic risk
Platelet count	Cellular haemostasis	Usually limited quantitative variation; may show modest delayed increase during recovery	May provide supportive but non-specific information on postoperative haemostatic adaptation	Does not adequately reflect platelet activation status
Inflammatory markers (e.g., CRP, leukocyte-related indices)	Inflammation–coagulation interface	Early postoperative increase; variable normalization thereafter	Support interpretation of persistent biological response after surgery	Poor specificity; limited value for individualized thrombotic risk prediction

## Data Availability

No new data were created or analyzed in this study.

## Abbreviations

The following abbreviations are used in this manuscript:

ADAMTS13	A disintegrin and metalloproteinase with thrombospondin type 1 motif, member 13
ADP	Adenosine diphosphate
AUA	American Urological Association
AUC	Area under the curve
CaP	Prostate cancer
CRP	C-reactive protein
DVT	Deep vein thrombosis
EAU	European Association of Urology
EC	Endothelial cell
NETs	Neutrophil extracellular traps
PAF	Platelet-activating factor
PAI	Plasminogen activator inhibitor
Pg	Plasminogen
Pn	Plasmin
RP	Radical prostatectomy
TGA	Thrombin generation assay
TP	Tissue pathway/tissue factor pathway
tPA	Tissue plasminogen activator
TXA_2_	Thromboxane A_2_
VTE	Venous thromboembolism
VWF	Von Willebrand factor
VWF:Ag	Von Willebrand factor antigen
VWF:CB	Von Willebrand factor collagen-binding activity
α_2_AP	Alpha-2 antiplasmin
